# Pneumomediastinum following a prolonged second stage of labor – an emphasis on early diagnosis and conservative management: a case report

**DOI:** 10.1186/s13256-017-1482-1

**Published:** 2017-11-06

**Authors:** Stephanie Whelan, Matthew Kelly

**Affiliations:** 1 Redlands Hospital, 54 Glen St, Kelvin Grove, QLD 4059 Australia; 2 Beaudesert Hospital, Beaudesert, Queensland Australia

**Keywords:** Pregnancy, Intrapartum, Esophageal rupture, Pneumomediastinum, Subcutaneous emphysema

## Abstract

**Background:**

Esophageal rupture is an extremely rare condition to occur to a pregnant or postnatal woman. Esophageal ruptures have been previously described in the literature; however, they are most common in the setting of hyperemesis gravidarum.

**Case presentation:**

This case report describes a 27-year-old white woman who began complaining of central chest pain and shortness of breath 3 hours after a normal vaginal delivery, with no history of vomiting antenatally or intrapartum. A chest X-ray and computed tomography pulmonary angiogram confirmed surgical emphysema and pneumomediastinum, and a diagnosis of esophageal rupture was made based on these findings. She was stable and conservative management was initiated; she improved over 4 days. Resolution of surgical emphysema was demonstrated on serial chest X-rays without requiring contrast swallow or surgical intervention.

**Conclusions:**

This case exemplifies the importance of a timely diagnosis of esophageal rupture in ensuring a positive outcome for the patient. Delay in diagnosis can lead to an increase in morbidity and mortality.

## Background

Esophageal rupture, especially in the absence of hyperemesis gravidarum, is extremely rare in pregnant women. In 1724, Dr Boerhaave first described a fatal esophageal rupture and it is thought to occur during or after persistent vomiting [1]. Approximately 200 cases of postnatal esophageal perforation have been reported globally [2]. It has been suggested that the incidence of subcutaneous emphysema originating from the mediastinum is approximately 1 in 100,000 deliveries [2]. This rare condition requires prompt diagnosis and treatment as failure to recognize the condition, and a delay in diagnosis, may lead to an increase in morbidity and mortality [3].

## Case presentation

### Patient information

A 27-year-old white woman, primigravida, presented at 36 weeks and 6 days’ gestation in spontaneous labor. Her antenatal course was uncomplicated.

She was fit and well with a body mass index (BMI) of 20. She did not smoke tobacco and identified as a Jehovah’s Witness. She had arranged a documented advanced health care directive in the event of an emergency. She had no episodes of vomiting antenatally. Her medical history included being investigated for palpitations for which she had previously had blood tests, including thyroid function and a Holter monitor, all of which were normal. She had been reviewed by the cardiology team who had arranged for a transthoracic echocardiogram to be performed postnatally.

### Clinical findings

On the day of delivery, she ruptured her membranes and went into spontaneous labor 1.5 hours later, taking 13 hours and 40 minutes of active labor to reach full dilation (Table 1). No episodes of nausea or vomiting were noted throughout the labor. She used nitrous oxide-oxygen gas mixture and non-pharmacological pain relief, including an oil diffuser and massage. Once she was in second stage, her contraction rate decreased to two in 10 minutes and an oxytocin infusion was commenced. After 2 hours and 22 minutes of active pushing she had a vaginal delivery of a live baby boy in good condition with Appearance, Pulse, Grimace, Activity, and Respiration (APGAR) scores of 9 at 1 minute and 9 at 5 minutes of age. The baby did not require any resuscitation and weighed 2980 g. Intramuscular oxytocin was administered and the placenta and membranes were delivered with controlled cord traction. No postpartum hemorrhage was noted.

**Table 1:** Timeline

Dates	Relevant past medical history and interventions		
5 Sep 2016	27-year-old, G1P0, currently 36+6 weeks pregnant, antenatal course uncomplicated. Jehovah’s witness, valid Advanced Health Directive. History of palpitations, investigated with thyroid function tests and Holter monitor, results normal. Patient awaiting echocardiogram		
Dates	Summaries from initial and follow-up visits	Diagnostic testing (including dates)	Interventions
6 Sep 2016	Central chest pain and shortness of breath. Tachycardic and subcutaneous emphysema noted in neck and clavicles. Diagnosis made of esophageal rupture	Chest X-ray (6 Sep 2016) and CTPA (6 Sep 2016) revealed pneumomediastinum and subcutaneous emphysema	Treated conservatively. Moved to the high dependency unit, given supplemental oxygen, intravenously administered fluids, intravenously administered antibiotics
7 Sep 2016–8 Sep 2016	Follow-up: • patient’s symptoms resolved • patient compliant with treatment, nil adverse events	Serial chest X-rays daily 6 Sep 2016–8 Sep 2016	Intravenously administered antibiotics regime changed to cover pneumonitis. Gradual return to oral intake

*CTPA* computed tomography pulmonary angiogram, *K*

Three hours post-delivery she began to complain of central chest pain, which was constant in nature, non-radiating, and associated with shortness of breath on standing. She had become tachycardic to 120 to 145 beats per minute (bpm); however, all other observations were normal with a stable blood pressure of 130/75 mmHg, oxygen saturations of 98% on room air, respiratory rate of 18 breaths per minute, afebrile, with no evidence of cyanosis or calf swelling. On examination, subcutaneous emphysema was palpable over her clavicles and into her neck, with good air entry bilaterally.

### Diagnostic assessment

**Fig. 1:**
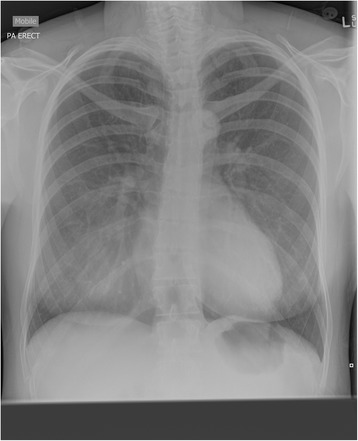
Chest X-ray demonstrating subcutaneous emphysema and pneumomediastinum

An urgent chest X-ray was requested as well as an urgent review by the medical registrar on call for the evening, in addition to hematological studies and an electrocardiogram (ECG). The ECG showed sinus tachycardia. An arterial blood gas was attempted, although, unfortunately, it was found to be a venous blood sample with a pH of 7.416, partial pressure of oxygen (pO_2_) 47 mmHg, lactate 4.59 mmol/L, and bicarbonate (HCO_3_) 19.6 mmol/L. The chest X-ray (Fig. 1) revealed evidence of pneumomediastinum with subcutaneous emphysema at the base of her neck, no visible pneumothorax. A diagnosis of esophageal rupture was made based on the findings of the chest X-ray. She was then discussed with the on-call physician, obstetric medicine, and retrieval services to discuss her management and transfer. A plan was made for a computed tomography pulmonary angiogram (CTPA), which excluded a pulmonary embolism and confirmed pneumomediastinum and subcutaneous emphysema within our patient’s neck and right chest wall, with no pleural effusions or pneumothorax.

Other causes for the subcutaneous emphysema were considered prior to making a diagnosis. The absence of any trauma either blunt or penetrating and with no history of foreign body aspiration, external trauma was excluded as a cause of our patient’s surgical emphysema and pneumomediastinum. Subcutaneous emphysema can also be a complication when a patient has a pneumothorax. This important cause was annulled on the serial chest X-rays and CTPA, which also allowed us to exclude embolic, infective, and cardiac causes with the addition of bloods tests and ECG. Barotrauma caused by the forced expiration during the second stage of labor may have ruptured alveoli or small pleural blebs, and caused the pneumomediastinum and surgical emphysema to occur. Decreasing the likelihood of barotrauma and alveolar rupture as the primary cause of the X-ray findings was the fact that our patient denied any personal or family history of lung disease, alpha-1 antitrypsin deficiency, or collagen disease which would predispose her to such trauma. She also reported no history of alveolar rupture or spontaneous pneumothorax. In the absence of a significant tobacco smoking history and other risk factors, the decision was made that the prolonged straining during labor most likely contributed to an esophageal rupture as the primary cause for our patient’s symptoms and pathology. Given the known high mortality associated with esophageal rupture, albeit rare, a decision was made on the X-ray findings; the prolonged second stage of labor and associated straining being most likely suggestive of esophageal rupture and timely treatment should ensue.

### Therapeutic intervention

The investigation findings were discussed with an obstetric medicine physician who advised that as our patient was hemodynamically stable the likely cause was an esophageal rupture and to treat as such immediately. A consensus was reached that a contrast swallow or upper gastrointestinal endoscopy would only be required if our patient did not clinically improve with treatment or became hemodynamically unstable. She was moved to our high dependency unit and treatment for esophageal rupture was commenced, which included her being kept nil by mouth, supplemental oxygen via nasal prongs, intravenously administered fluids, and intravenously administered benzylpenicillin. The intravenously administered antibiotics were changed to intravenously administered piperacillin/tazobactam after one dose postpartum to cover for possible pneumonitis. Anesthetic input was provided and she was commenced on buprenorphine. Her pain settled after 24 hours and subsequent X-rays showed no extension of pathology. She responded to treatment and thus the opinion was that the correct diagnosis had been made based on the clinical picture, chest X-ray, and CTPA. As she remained stable and responded to treatment, the recommendation from the obstetric medical physician was that a contrast swallow or gastrointestinal endoscopy would not change management and thus was not required.

### Follow-up and outcomes

She was regularly reviewed and had a gradual return to oral intake with clear fluids, thicker fluids, and then solids over 48 hours. She continued to improve and was discharged on day 4 postpartum. She was informed about the importance of notifying future care providers of her previous esophageal rupture so that provisions could be instituted in future pregnancies in order to minimize the risk of reoccurrence.

## Discussion

Spontaneous esophageal ruptures are rare, especially in young pregnant women and are usually associated with a history of hyperemesis gravidarum. The diagnosis is suggested by the classic Mackler’s triad: vomiting, chest pain, and subcutaneous emphysema [1, 4]. This triad can be rare however, with 20 to 45% of patients having no history of vomiting and with clinical features that can vary greatly depending on the site of the perforation [5]. As such the initial diagnosis is only made in one third of cases [6]. Boerhaave’s syndrome most commonly presents in older males secondary to excessive alcohol consumption, neurological disease, severe esophageal burns, and scleroderma [7]. Vomiting is a known predisposing factor in the published case reports of spontaneous esophageal perforations in first trimester, late pregnancy, and postpartum, with only one other case report documenting a spontaneous esophageal rupture in pregnancy in the absence of any vomiting antenatally or intrapartum [6]. There are differing opinions in the literature regarding the effect of a long labor on the risk of esophageal perforation, which may have been relevant in this case. One of the earliest published reviews of surgical emphysema in labor was by Gordon (1927) who looked at 130 patients and found that there was an increased risk in healthy primigravid women with a long labor [8]. This was also observed by Wozniak and Blackburn, who described a 20-year-old primigravida who had slow progress and 2.5 hours of active pushing in the absence of any nausea or vomiting [2]. It was the prolonged active pushing that was postulated to cause the patient’s symptoms. By contrast however, a more recent study found no significant correlation between the duration of labor and the likelihood of developing surgical emphysema [9].

In the absence of other predisposing factors it is most likely that the injury occurred in the second stage of labor due to prolonged pushing (2 hours and 22 minutes). The mechanism of pushing causes straining against a closed cricopharyngeus muscle, and increased abdominal pressure then causes an acute rise in esophageal and intra-alveolar pressure [6]. The Valsalva maneuver during forced expiration is repeated constantly during pushing causing alveolar barotrauma. Air then travels from the damaged alveoli through sheaths to the mediastinum and into the subcutaneous tissue [7].

The successful outcome of the case was directly related to the early diagnosis of the surgical emphysema and pneumomediastinum with a chest X-ray. Findings diagnostic of a spontaneous esophageal rupture are seen in 97% of cases. Abnormalities on the chest X-ray can include subcutaneous emphysema, pneumothorax, hydropneumothorax, basal consolidation, or pneumomediastinum effusion [2, 6]. The location of the perforation determines the clinical features: an anterior perforation would see significant surgical emphysema as in this case. If the chest X-ray had shown more involvement of the lungs and pleural cavity, this would be more suggestive of a posterior or lateral perforation [6]. Investigation with a CTPA in this case did not add information to the diagnosis; however, it did help to exclude a pulmonary embolism from the differential diagnosis. Further radiological evidence by means of a swallow radiograph can help to locate and determine the severity of the tear, but its role in initial diagnosis is limited and can delay prompt management of the issue. If performed, a water-soluble contrast such as Gastrografin is preferred as barium may cause severe mediastinitis [1]. Multiple reports that have used contrast radiography either at the time of spontaneous injury or in the days that followed failed to show a leak from the esophagus [2, 4, 6]. This is likely due to either the size of the perforation or the spontaneous closure of the injury due to appropriate and timely management. The use of an endoscopy to confirm the diagnosis is not routinely recommended. It has been previously reported as not adequately sensitive and in the context of esophageal disease could be potentially harmful [10, 11].

The prompt diagnosis and correct management of the esophageal perforation played a pivotal role in the successful outcome for this patient. The principles of management remain controversial and can be either conservative or surgical. In deciding between conservative and surgical approaches the site/size of the rupture, degree of mediastinal contamination, and time from diagnosis should all be considered [5]. Conservative management, as in this case, is aimed at preventing further mediastinal contamination by withholding oral intake, giving broad spectrum antibiotics, and employing nasogastric decompression when indicated [5]. Surgical management is generally required when there has not been an early diagnosis, as patients with esophageal perforation become compromised quickly. It involves drainage of mediastinal or pleural collections, repair of the defect, or stent insertion [7]. Complications are life-threatening and include tension pneumothorax, further leaks, acute mediastinitis, mediastinal sepsis, and multiple organ failure, all of which can begin to occur within a few hours of injury [7, 11]. Early diagnosis is the key to a successful outcome with research showing that if treatment is commenced in the first 24 hours, the survival rate is 75%. After 24 hours the survival rate falls to 35% and if treatment is commenced after 48 hours, the survival rate is between 11 and 25% [11]. Due to the rarity of the condition there is currently no data available on the likelihood of recurrence in future pregnancies or later in life.

## Conclusions

Esophageal perforation during labor is an extremely rare but life-threatening condition. This case exemplifies how early diagnosis and prompt correct management is the key to a successful outcome. Most esophageal perforations can be diagnosed based on the clinical history, clinical examination, and chest X-ray findings. It is important to understand that if further investigations are conducted they should not delay treatment.

## Abbreviations

CTPA: Computed tomography pulmonary angiogram
ECG: Electrocardiogram
